# The scientific challenge of the 21^st ^century: from a reductionist to a holistic approach via systems biology

**DOI:** 10.1186/1471-2202-7-S1-S1

**Published:** 2006-10-30

**Authors:** Rita Levi-Montalcini, Pietro Calissano

**Affiliations:** 1 EBRI Foundation and Institute of Neurobiology and Molecular Medicine, CNR, Roma, Italy

In the 20^th ^century the study of life was generally approached with a reductionistic approach: analysing the simple and extrapolating to more complex systems. Thanks to this strategy the most important functions of a living organism have been dissected and elucidated: the structure of DNA, the complex machinery subserving the synthesis of proteins, the mechanism for the utilization of food and the generation of energy. On the therapeutic side, the discovery of sulphamides and antibiotics, of specific vaccines directed against the most dangerous diseases or the synthesis of tranquillizers and anti-psychotics in the field of neurology and psychiatry, provided the basis for a modern, socially oriented medicine. The paradigm for such a strategic approach to biology was the "dogma" proposed by Tatum and Beadle: one gene, one protein, one function.

In the twenty first century we know that this, as well as many other biological dogmas, are obsolete or are doomed to die. Thus, for instance, it is presently a well established notion that one gene may give rise to several proteins and each of these proteins may undergo several post-translational modifications conferring distinct functional activities. These findings, in turn, provide a general clue as to why, although the human genome has a number of genes similar to those of a worm such as *C. elegans*, yet the number and versatilities of proteins and corresponding functions are incomparable in the two organisms. Thus, each gene could be compared, to some extent, to a sort of kaleidoscope; as it is sufficient to change the view of a kaleidoscope by slightly moving its position, the expression of a gene into a given functional protein entity may be modulated in a variety of functions according to the developmental stage, cell type, and needs in relationship with environment.

These findings and the impressive, often revolutionary technical achievements of the last two decades, induced experimental scientists and theoretical biologists to device novel strategies and epistemological approaches.

We wish to briefly discuss, as an example, the evolution of the studies on NGF (nerve growth factor) and eventually try to extrapolate these short considerations to biology at large in the 21^st ^century.

In the fifties and subsequent few decades, the discovery by one of us (RLM) of this growth factor [1,2], followed by the characterization of its functional properties [3-6] was a typical fruit of a reductionistic success: identifying a single substance, demonstrating its diffusible nature and showing its specific function of inducing the differentiation of two types of neurons constitutive of sympathetic and sensory ganglia. The subsequent experimental attempt was again a typical case of a reductionistic success: devising an *in vitro *culture system, to replace the complex and time consuming *in vivo *experiments carried out in chick embryo, in order to assess the presence of this "nerve growth promoting activity" – subsequently identified as nerve growth factor (NGF) – primarily in sarcoma and subsequently in tissues and biological fluids. This attempt ended with an *in vitro *bioassay which, within 18–24 hours, allowed to detect NGF activity evidenced by an impressive halo of nerve fibers. This was an even more stringent case of a successful reductionistic approach.

In the last decade of the past century the studies on NGF took a route which presently induces a growing number of laboratories to carry out systemic, "holistic" strategies. Thus, for instance, the properties of NGF receptors, endowed with completely different structure, functional properties and mechanism of action – to the extent, for instance, that they may exert both pro and anti-apoptotic actions via activation of a plethora of intracellular pathways – [7,8] need today a novel approach to answer questions such as: how many different genes are involved in such a complex pattern of activities, considering, for instance, that an anti-apoptotic pathway generally involves hundreds of different genes and proteins? In order to put together in a unique, comprehensible picture, all the data that are emerging in this field of investigations, a "global" approach with highly sophisticated techniques is mandatory.

On the other side, the broadening of studies on NGF target cells, which in the first 2–3 decades after its discovery were confined to sensory and sympathetic neurons, to cholinergic and possibly other neurons of the central nervous system, followed by the demonstration that several other cell types belonging to the endocrine and immune systems are target of NGF action, also requires a systemic strategy, to frame the whole populations of target cells into an "organismic" view of NGF functions and mechanism of action [9].

As mentioned above, in the 21^st ^century, also due to the realization of extremely sophisticated and powerful techniques, such as the possibility of cloning any gene, analysing the expression of thousands of genes and of the corresponding coded proteins within the context of a specific cellular function, investigating the whole complex machinery linking energy metabolism to modulation of gene expression, neurobiologists must adopt novel strategies and technical procedures. The term that summarizes such an approach is "systems neurobiology" and the metaphor that better fits with such systemic strategy is that a single protein is to a neuron as a neuron is to whole brain. Accordingly, both for analysing a neuron – which is constituted by thousands of proteins – and for studying the function of brain – composed of billions of neurons -, it is mandatory to resort to proper techniques such as genomics, proteomics or transcriptomics, which, by definition, involve the contemporaneous analysis of hundreds or thousands of genes and/or of the corresponding coded proteins or metabolic products. Thus, considering the wholeness of cellular systems, adopting proteomics or functional genomics attacks might be as important for our understanding of brain functions as, for example, the wholeness of economical markets is to the study of macroeconomics.

From this point of view this volume is a living testimony of a novel attack to neurobiological problems, and a variety of extremely interesting problems constitute the bulk of the assay. The modular system approach to investigate the mechanisms involved in apoptotic processes; the compelling need to elucidate how mRNAs and proteins are addressed to their specific sites of action along axons and dendrites up to their final destination at synaptic structures; how these polymeric structures act as multi-sensor devices of neurotransmission and the way the storage of synaptic activities can be exchanged and integrated to provide memories into a larger scale; and, last but not least, the role played by non coding RNA in brain. All these topics are dealt with in the volume with interesting and novel findings and hypothesises.

Another problem of great impact deals with parameter estimation in signalling pathway modelling, and with how enzyme kinetics and metabolic fluxes in general contribute to the control of cell operation. Computational frameworks able to integrate data from multiple sources are discussed as a tool for unravelling the biological mechanisms underlying the regulation of gene expression. Finally, a particular welcome is to be addressed to the long lasting case of mathematics dealing with biological problems. The entry of this discipline into the biological scene was in past decades discouraged by the paucity of data and by the even more remote possibility of analysing them in a comprehensive fashion. For this reason a theoretical biology was a hazardous task and the few attempts involving this discipline were generally unsuccessful. Nowadays, these drawbacks can be overcome by the impressive collection of data available on a specific experimental issue and by the availability of extremely powerful computers. Thus, the mathematical modelling of neuron development and of signal transduction pathways that are discussed in two distinct chapters may offer new vistas to the biological landscapes. The last article shows how the coming of age of computational systems neurobiology entails the need of automated support to handle formal models and of developing standard and guidelines to maximise the diffusion of its scientific production.
